# Machine Learning‐Enhanced Clinical Decision Support for Diagnosing Sinusitis With Nasal Endoscopy

**DOI:** 10.1002/alr.70045

**Published:** 2025-10-15

**Authors:** Dipesh Gyawali, Thomas Mundy, Majid Hosseini, Morteza Bodaghi, Akio Fujiwara, Sejal Shyam Bhatia, Kayla Baker, Elena Bartolone, Dhara Patel, Henry Chu, Raju Gottumukkala, Jonathan Bidwell, Edward D. McCoul

**Affiliations:** ^1^ Department of Otorhinolaryngology Ochsner Health New Orleans Louisiana USA; ^2^ Ochsner Clinical School University of Queensland New Orleans Louisiana USA; ^3^ Informatics Research Institute University of Louisiana at Lafayette Lafayette Louisiana USA; ^4^ Department of Otolaryngology–Head and Neck Surgery Tulane University School of Medicine New Orleans Louisiana USA

**Keywords:** artificial intelligence, diagnosis, machine learning, nasal endoscopy, rhinosinusitis

## Abstract

**Background:**

Sinusitis is a prevalent disease for which nasal endoscopy (NE) is an optimal diagnostic modality. However, NE accuracy is limited by inter‐operator variability in landmark identification and localization of mucus that is necessary for sinusitis diagnosis. We sought to develop a novel multi‐class machine learning (ML) framework that detects anatomical landmarks and structures for sinusitis assessment as supported by clinical best practices.

**Methods:**

A total of 3513 NE images from 452 patients were manually annotated by four physicians for three classes: middle turbinate (MT), inferior turbinate (IT), and mucus. A YOLOv11‐nano model was trained for multi‐class detection and segmentation. We developed a rule‐based logic for middle meatus localization, implementing a clinical algorithm that applies anatomy Intersection over Union (IoU) and conditional logic for sinusitis diagnosis. The system was validated on 178 images from 50 patients with chronic rhinosinusitis without polyps (CRSsNP) with benchmarking of real‐time performance.

**Results:**

The multi‐class detection and segmentation model achieved > 75% F1 score for detecting turbinates with mucus. The clinical algorithm achieved 75.0% sensitivity, 76.0% specificity, and 75.2% accuracy for sinusitis classification, with a F1 score of 81.8%, approaching the accuracy of a trained otolaryngologist. The framework achieved near real‐time performance at > 20fps on GPU device, demonstrating suitability for integration into live clinical workflows.

**Conclusion:**

This novel ML‐driven diagnostic framework with a rule‐based clinical algorithm enhances decision‐making for diagnosing sinusitis with NE. By reducing inter‐operator variability, achieving performance comparable to otolaryngologists, and enabling real‐time processing for non‐specialists, this work holds potential for standardizing care and improving patient outcomes. Future research will focus on expanding to different sinusitis phenotypes and prospective real‐time implementation in clinical settings.

## 1 Introduction

Nasal endoscopy (NE) plays a crucial role in the diagnosis of sinusitis. Conventional diagnostic methods such as computed tomography (CT) have historically encountered challenges due to frequently failing to capture pathological changes, resulting in instances where abnormalities detected during NE are missed on imaging [1, 2, 3]. These limitations highlight NE as a reliable diagnostic tool given its use during nasal exams to evaluate sinus conditions such as chronic rhinosinusitis (CRS) and perform surgical interventions [4, 5, 6]. Advancements in NE technology such as video quality and maneuverability have expanded the potential of NE in the detection of increasingly subtle features, and continued advances in clinical support software along with artificial intelligence (AI) and machine learning (ML) provide opportunities to aid in the classification and interpretation of nuanced NE findings [7]. These improvements have the potential to increase the diagnostic value of NE findings and decrease the utilization of imaging modalities like CT, which could result in expanding access to care by decreasing costs with more effective management of sinonasal conditions [4, 7].

Despite these advances, integration of ML into clinical endoscopy remains challenging due to the high variability in nasal anatomy, lack of standardized protocols, and absent criteria for distinguishing between purulent and non‐purulent mucus. Variability in nasal anatomy can lead to significant inter‐rater discrepancies in assessing features such as mucosal changes, inflammation, and mucus discharge [8, 9, 10]. Moreover, the lack of standardized protocols for NE further confounds diagnostic variability, allowing minor signs of sinonasal pathology to be overlooked even by experienced clinicians [9, 10]. While prior studies have highlighted the potential benefits of ML in improving diagnostic accuracy, the current integration of these technologies into NE is limited, necessitating further research to overcome these challenges [11, 12]. Considering the outstanding performance of ML when determining anatomic and pathological features in recorded images and video [13], ML as a tool for supporting the interpretation of NE findings could potentially have a meaningful role in medical practice. There has been progress, but studies indicate that the integration of ML and NE is limited due to the need for further research [11, 12].

This study aims to develop and validate an ML framework that detects landmarks and features for sinusitis assessment by localizing the middle meatus (MM) and analyzing for purulent mucus. The detection of purulent mucus in the MM represents an objective endoscopic finding indicating active sinonasal inflammation, which may vary temporally and spatially even within patients with established CRS. Establishment of this framework will support ML models that can be used to implement clinical best practices, help automate diagnosing sinusitis, and standardize future care in a manner that is on par with a trained otolaryngologist.

## 2 Materials and Methods

### 2.1 Dataset Description

A total of 3513 NE images (1024 × 768 px, JPEG) from 452 patients were obtained by a single physician (E.D.M.) during NE examinations on a Telepak system using a flexible video endoscope (11101VN, Karl Storz, Tuttlingen, Germany) equipped with photo capture buttons in a tertiary rhinology clinic at the Ochsner Medical Center in New Orleans, Louisiana, between September 2014 and February 2025. The calibration for white balance, angle adjustment, and light intensity were performed in a standardized fashion by the same individual before capturing the images during the examination.

We applied the following inclusion criteria to ensure image quality for model training: (1) images had minimal or no distortion, obstruction, clouding, or artifacts; (2) the lens was not obstructed by mucus or saliva; and (3) images were clear, non‐bloody, and interpretable. Images that failed to meet these criteria were excluded from the dataset.

Four physicians manually annotated the images for three classes: middle turbinate (MT), inferior turbinate (IT), and mucus of any morphology. Each image could contain multiple segmentation masks representing different anatomical structures and mucus deposits. We used the open‐source CocoAnnotator labeling software (https://github.com/jsbroks/coco‐annotator) for annotation. Inter‐rater reliability was assessed using Intersection over Union (IoU) as the evaluation criterion, with all annotations cross‐validated against a single expert labeler to ensure >80% F1 Score agreement.

### 2.2 Study Designs and Patients

This retrospective study analyzed NE images from patients with either normal sinonasal findings or CRS without nasal polyps (CRSsNP). The overall dataset included both normal and pathological cases for training the multi‐class detection and segmentation model.

For validation of the clinical algorithm, we conducted a chart review to identify 50 patients with confirmed CRSsNP from the senior author's clinical practice. CRSsNP diagnoses were verified through CT scans and corresponding clinical data mapped to NE. This subset generated an independent test set of 178 images, which was used to compare ML framework performance against otolaryngologist expert diagnoses. Of the 178 images, 132 (74.2%) were classified as positive for sinusitis signs and 46 (25.8%) as negative based on expert otolaryngologist assessment. This distribution reflects the expected prevalence of active inflammatory findings in a tertiary rhinology practice. Each image was independently labeled by an expert otolaryngologist for the presence or absence of sinusitis signs visible in that specific endoscopic view, regardless of the patient's overall clinical diagnosis. This image‐level annotation approach resulted in both “positive” and “negative” classifications within the CRSsNP patient cohort, reflecting the variable presentation of mucosal findings across different anatomical regions and disease activity phases. Performance metrics were calculated using a confidence threshold of 0.3. In addition to single‐point metrics at the 0.3 IoU threshold, we conducted ROC curve analysis across all possible thresholds to comprehensively characterize algorithm performance and validate threshold selection. Patients diagnosed with chronic rhinosinusitis with nasal polyps (CRSwNP) were excluded from this study.

### 2.3 Dataset Allocation and Model Development

From the total dataset, we employed an 80:20 split for training and validation, respectively. An independent test set was reserved to evaluate the clinical algorithm performance. This separation prevented data leakage and reduced potential bias, ensuring that the test set represented real‐world clinical scenarios for sinusitis diagnosis.

We selected YOLOv11‐nano for multi‐class detection and segmentation of anatomical landmarks and structures in NE images. This model was chosen for its efficiency and capability to achieve real‐time performance while maintaining high accuracy [14]. The model was trained to simultaneously detect and segment the three classes of MT, IT, and mucus.

The test set included complete annotations for all three classes, along with clinical metadata including laterality (left/right) and ground truth sinusitis diagnoses from an expert otolaryngologist. Ground truth diagnoses were recorded by annotating NE images “Positive” for sinusitis in cases with an established history of CRS that were imported from electronic patient records sans identifying information. This comprehensive annotation enabled evaluation of both the segmentation performance and the clinical algorithm's diagnostic accuracy compared to otolaryngologist assessment.

### 2.4 Clinical Algorithm

We developed a rule‐based clinical algorithm that implements best practices for sinusitis diagnosis using multi‐class ML detection and segmentation. The algorithm operates through three stages: (1) multi‐class detection and segmentation, (2) MM localization using rule‐based logic, and (3) sinusitis diagnosis using conditional logic based on anatomy IoU.

1. Multi‐class detection and segmentation
  The YOLOv11‐nano model simultaneously detects and segments two landmarks and one structure: MT, IT, and mucus. The model outputs bounding boxes and segmentation masks for each detected class with confidence scores ranging from 0 to 1.
2. Rule‐based logic: Middle meatus localization
  Since the MM is not directly visible in a subset of NE images due to anatomical positioning and occlusion, we developed geometric rules to localize the MM region using detected MT/IT landmarks:
  (a) Primary method (MT + IT):
    (i) Boundary extraction: Edge coordinates were extracted from the segmentation masks of MT and IT using segmentation contours.
    (ii) Medial boundary identification:
      - For right nasal cavity, the leftmost edge of the MT segmentation mask defined the medial boundary.
      - For left nasal cavity, the rightmost edge of MT segmentation mask defined the medial boundary.
    (iii) MM region calculation:
      - The MM bounding box was computed as:
$$MM_{\mathrm{box}}=\left\{\begin{array}{c} \;x_{1}={x_{\mathrm{MT}}}_{\mathrm{edge}\;}\;,\\ \;y_{1}=\max\left({y_{\mathrm{MT}}}_{\mathrm{top}}-\;\Delta y,\;0\right)\;,\\ \;y_{2}={y_{\mathrm{IT}}}_{\mathrm{top}}\;\left(\mathrm{fallback}:{y_{\mathrm{MT}}}_{\mathrm{bottom}}\right.\\ \left.\;\;+\;0.5h_{\mathrm{MT}}\;\mathrm{if}\;\mathrm{IT}\;\mathrm{undetected}\right), \end{array}\right.$$
        where:
        - Δx=50px,Δy=20px (empirically derived offsets to capture the MM region
        - $h_{\mathrm{MT}}$ denotes the height of MT segmentation mask.
        - *x* represents the *x* ‐ coordinate value and y represents the *y* ‐ coordinate value.
  (b) Fallback method (MT only):
    When IT was not detected (cases include obstruction, poor image quality, or IT not in the frame), the MM region was estimated using MT morphology only.
    - The superior and inferior edges of MT segmentation mask define vertical boundaries.
    - Horizontal boundaries were calculated as:
$$x_{\mathrm{start}}={x_{\mathrm{MT}}}_{\mathrm{edge}}\;-\Delta x_{\mathrm{safe}},\;\;x_{\mathrm{end}}={x_{\mathrm{MT}}}_{\mathrm{edge}}\;,$$
      where $\Delta x_{\mathrm{safe}}=30\;px$ represents a conservative safety margin to avoid overestimation.
      The offsets (Δ*x*, Δ*y)* were calibrated using 50 manually annotated MM regions from the training set. Safety margins (Δ*x*_safe_) were designed to align with clinical guidelines for MM assessment in chronic rhinosinusitis.
3. Conditional logic for sinusitis detection framework
  The clinical algorithm applies conditional logic based on anatomy IoU to diagnose sinusitis.
  (a) Primary diagnostic pathway:
    This pathway calculates IoU between computed MM region and detected mucus as:
$$\mathrm{Io}U_{\mathrm{MM}-\mathrm{mucus}}=\frac{\mathrm{Area}\left({\mathrm{MM}}_{\mathrm{box}}\cap \mathrm{Mucu}s_{\mathrm{box}}\right)}{\mathrm{Area}\left({\mathrm{MM}}_{\mathrm{box}}\;\cup \mathrm{Mucu}s_{\mathrm{box}}\right)}$$
    A threshold of IoU > 0.3 triggers a positive sinusitis diagnosis.
  (b) Fallback diagnostic pathway:
    This pathway activates when MM localization fails. It calculates IoU between MT and mucus as sometimes substantial amount mucus is deposited over MT.
$$\mathrm{Io}U_{\mathrm{MT}-\mathrm{mucus}}=\frac{\mathrm{Area}\left({\mathrm{MT}}_{\mathrm{box}}\;\cap \mathrm{Mucu}s_{\mathrm{box}}\right)}{\mathrm{Area}\left({\mathrm{MT}}_{\mathrm{box}}\;\cup \mathrm{Mucu}s_{\mathrm{box}}\right)}$$
    A threshold of IoU > 0.3 triggers a positive sinusitis diagnosis.

**FIGURE 1:**
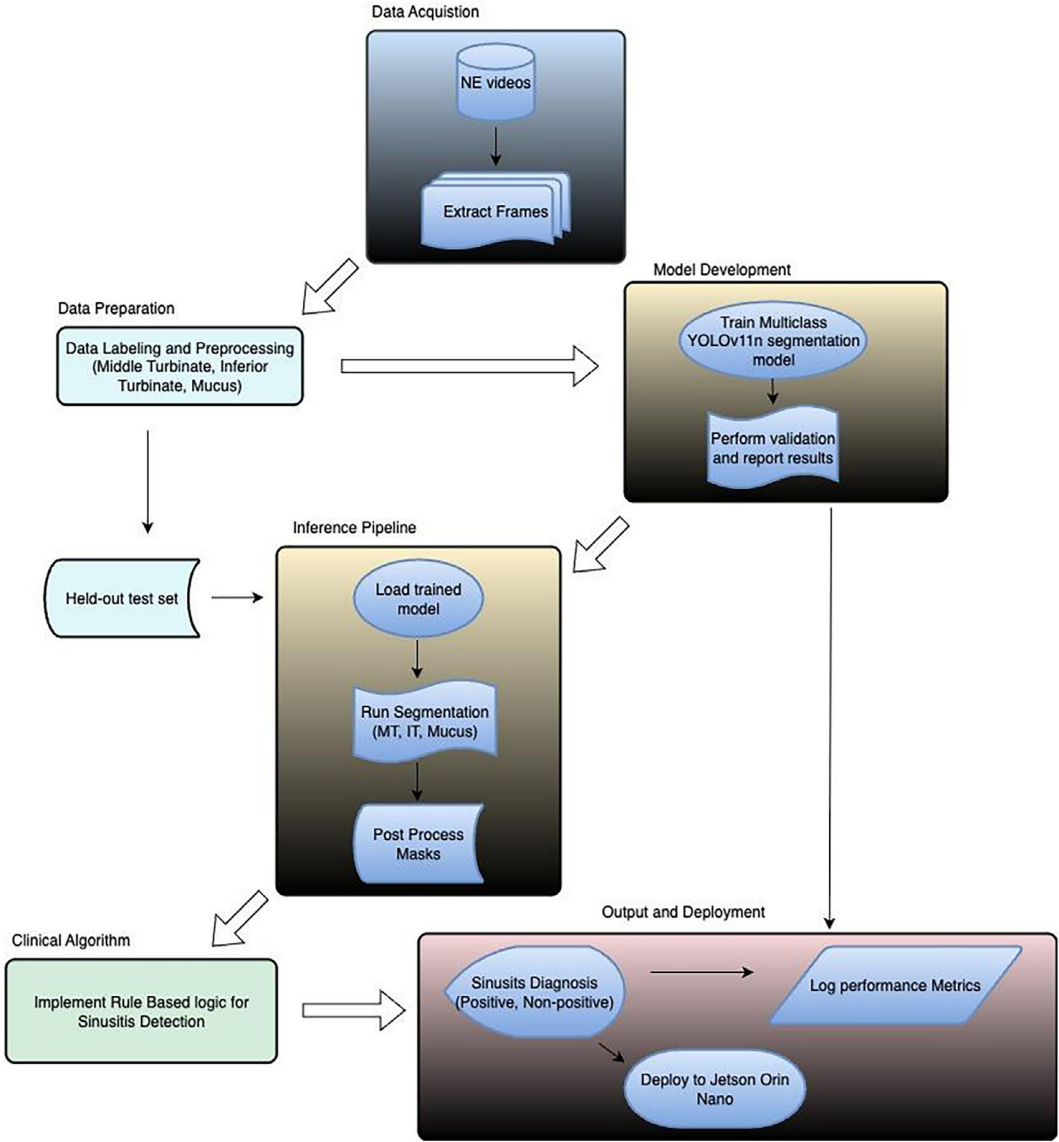
Process flow diagram for sinusitis diagnosis.

This rule‐based approach with conditional logic ensures diagnostic consistency while accommodating anatomical variations and imaging limitations commonly encountered in clinical practice. The fallback pathway (MT‐Mucus) was invoked when MM localization failed or was unreliable due to anatomical occlusion. Figure 1 demonstrates the overall process of our design.

### 2.5 Reference Standard

The reference standard consists of manually contouring landmarks and structures, and labels were assigned by a fellowship‐trained rhinologist (E.D.M.). Supplemental labeling was performed by three individual physicians who independently labeled a separate image subset without access to expert labels; only masks achieving IoU > 0.50 and F1 ≥ 0.80 were retained. Labeler‐expert agreement shows mean F1 = (0.84 ± 1.5) and Fleiss *κ* = 0.80, and the disagreements were resolved by consensus.

## 3 Results

### 3.1 Segmentation Performance

**FIGURE 2:**
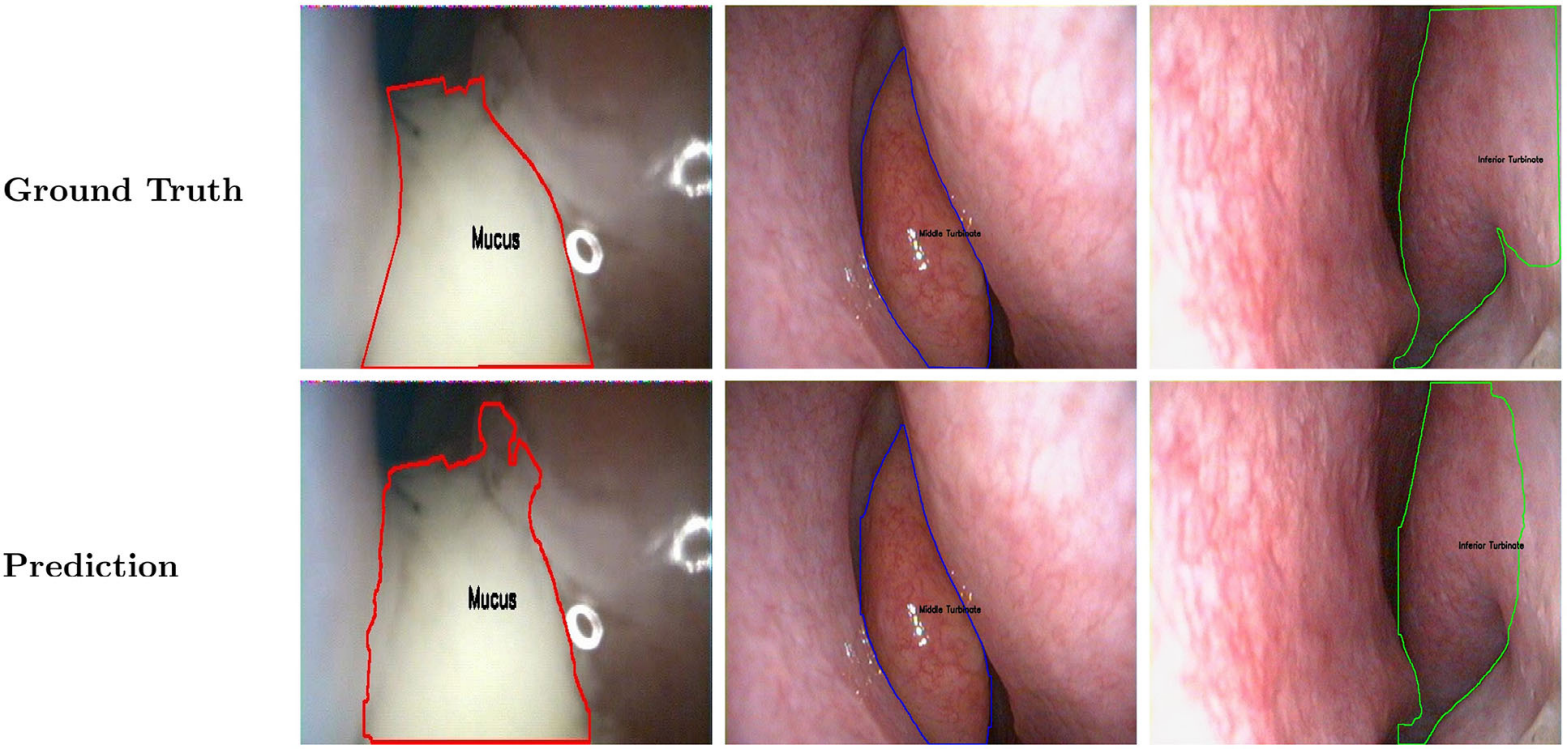
Ground truth and prediction for MT, IT, and mucus classes using YOLOv11‐nano model. Green boundary color represents IT, blue boundary color represents MT, and red boundary color represents mucus. One image represents only one class.

**FIGURE 3:**
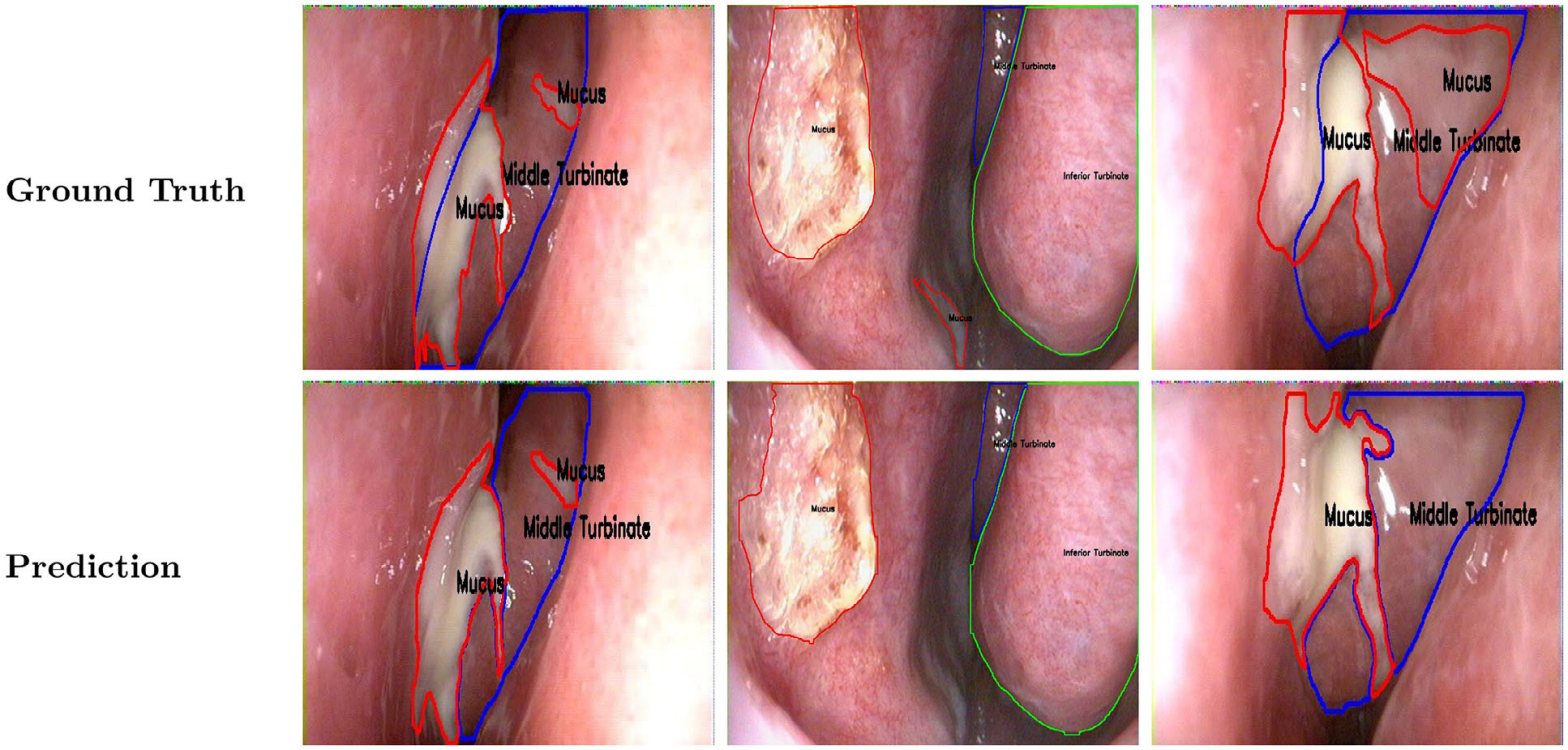
Ground truth and prediction for three classes using YOLOv11‐nano model (representative examples: multi‐classes in one image, middle meatus occluded, and variation in mucus structure). One image could represent multiple classes.

The YOLOv11‐nano segmentation model was trained on 80% of the dataset and evaluated on the validation set comprising 703 NE images (20% of the total 3513 images from 452 patients) with fivefold cross‐validation. The multi‐class detection and segmentation model achieved F1 scores above 75% for all three classes. The MT demonstrated an IoU of 0.94 and F1 score of 93.6%, the IT demonstrated an IoU of 0.86 and F1 score of 88.8%, and mucus segmentation achieved an IoU of 0.70 and F1 score of 78.2%. The overall average F1 score across all classes was 86.9%. Table 1 shows the overall performance across the validation set. Figure 2 shows segmentation on three classes using our framework where each image consists of only one class to segment. Figure 3 shows representative cases where the segmentation and detection are more challenging, such as multiple classes in one frame and instances of MM occlusion.

**TABLE 1:** YOLOv11‐nano segmentation performance on validation set.

Class	Total images	Total instances	IoU	Precision (%)	Recall (%)	F1‐ score (%)
Middle turbinate	511	561	0.94	95.1	92.2	93.6
Inferior turbinate	361	383	0.86	90.6	87.1	88.8
Mucus	376	759	0.70	85.0	72.3	78.2
Overall	703	908	0.83	89.3	84.8	86.9

### 3.2 Sinusitis Classification Performance

**FIGURE 4:**
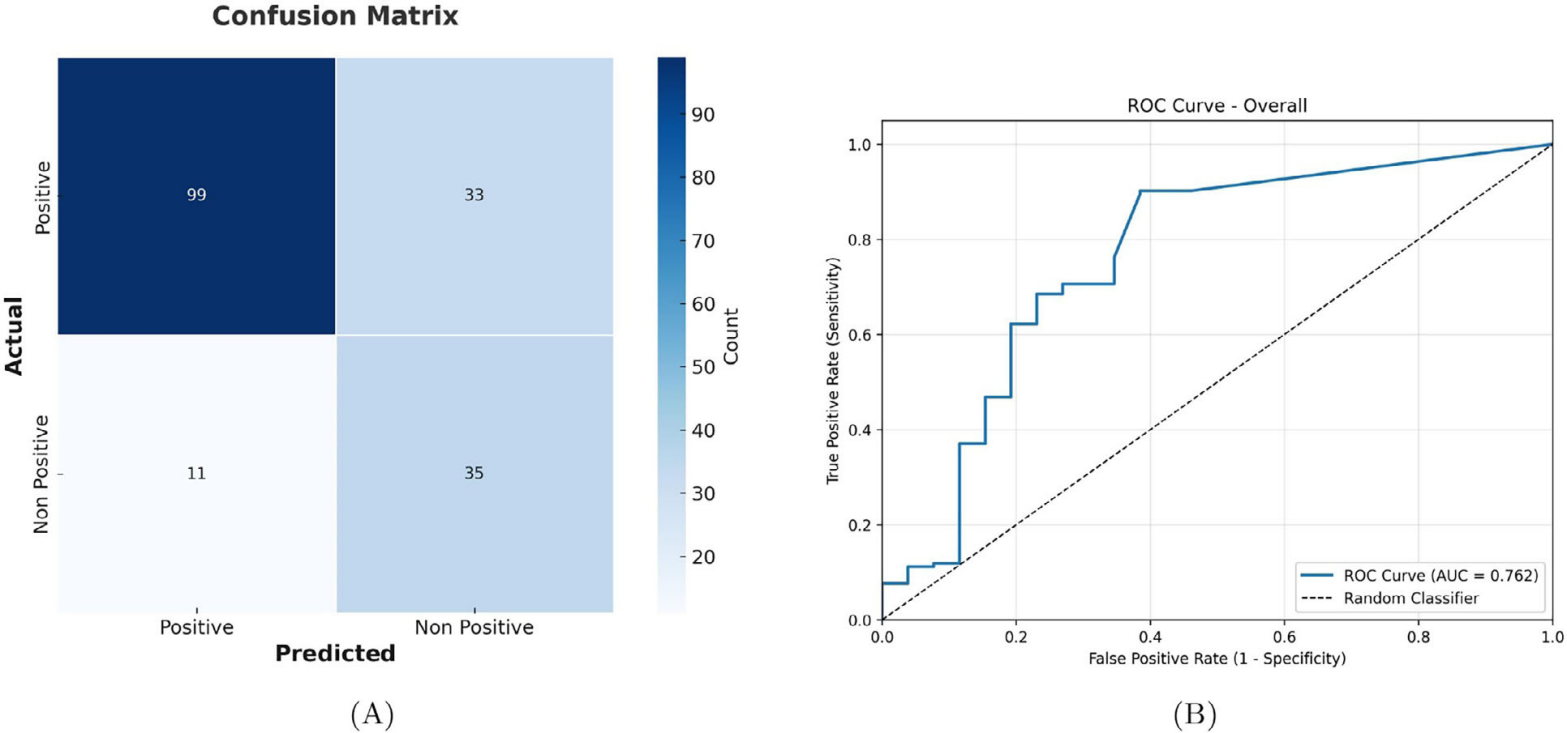
(A) Confusion matrix for sinusitis classification performance on test set (B) ROC curve for sinusitis classification.

For left nasal cavity images (*n* = 96), the algorithm achieved a sensitivity of 78.5%, specificity of 82.4%, and accuracy of 79.1%, with a precision of 95.4%. For right nasal cavity images (*n* = 82), performance was lower, with a sensitivity of 69.8%, specificity of 72.4%, accuracy of 70.7%, and precision of 82.2%. When pooling laterality, the overall diagnostic performance reached 75% sensitivity, 76% specificity, 75.2% accuracy, and precision of 90%. These results indicate strong performance for left nasal cavity images. Table 2 summarizes the classification performance metrics. The combined laterality metrics indicate a solid baseline for ML‑assisted sinusitis screening as shown in the confusion matrix in Figure 4A. Threshold sensitivity analysis across IoU values yielded an AUC of 0.762 (95% CI: 0.693–0.831), demonstrating good discriminative ability. The ROC curve in Figure 4B shows early sensitivity gains at lower thresholds with maintained specificity, validating our selection of 0.3 IoU threshold as an optimal operating point for clinical use.

**TABLE 2:** Classification performance for sinusitis diagnosis by nasal cavity laterality.

Region	Sensitivity (%)	Specificity (%)	Precision (%)	Accuracy (%)	F1‐score (%)
Left nasal cavity	78.5	82.4	95.4	79.2	86.1
Right nasal cavity	69.8	72.4	82.2	70.7	75.5
Overall	75.0	76.0	90.0	75.2	81.8

**FIGURE 5:**
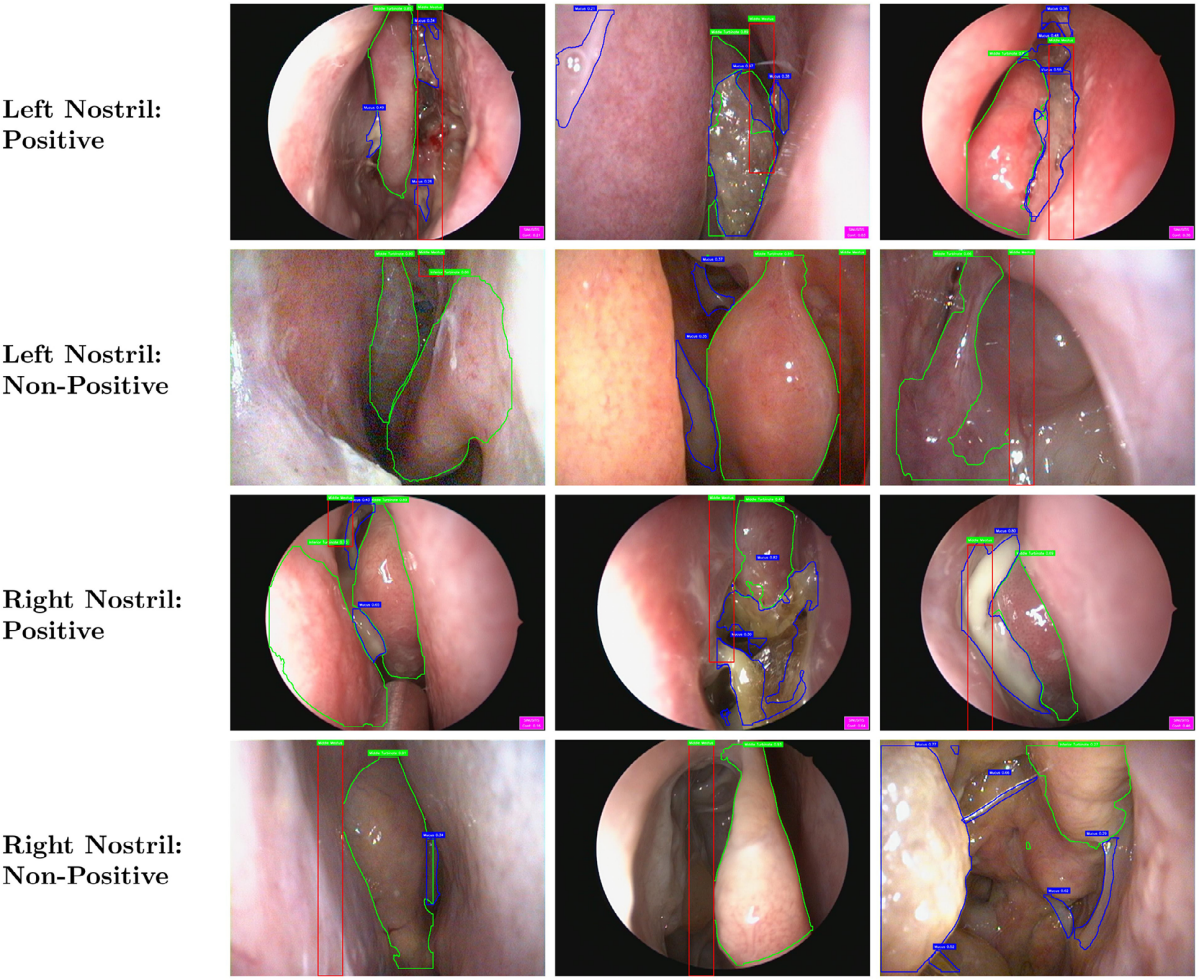
Sinusitis classification results on test set. Green boundary color represents MT or IT, blue boundary color represents Mucus, and red bounding box represents MM. The pink box at the bottom right corner triggers when sinusitis is detected.

To interpret how the clinical algorithm arrived at each diagnosis, we analyzed the trigger pathway used—either overlap between detected mucus and the MM (MM‐Mucus) or overlap with the MT (MT‐Mucus). For positive left‐sided images (*n* = 62), the MM‐Mucus trigger was used in 30 images and MT‐Mucus in 32 images. Several images satisfied both criteria. In non‐positive left‐sided images, two false positives were triggered by MT‐Mucus and a single false positive by MM‐Mucus. For positive right‐sided images (*n* = 37 detections), MT‐Mucus was responsible for 31 detections, and MM‐Mucus was responsible for six detections. Among non‐positive right‐sided images (*n* = 8), seven false positives were observed, triggered primarily by MT‐Mucus and one by MM‐Mucus. These findings highlight the importance of robust fallback logic for cases with incomplete visualization and support continued refinement of MM localization. Table 3 summarizes the trigger methods for sinusitis classification in test set. Pathway‐specific analysis revealed differential performance between diagnostic approaches. The MM‐Mucus pathway demonstrated higher precision (94.7%) compared to the MT‐Mucus pathway (87.5%). Despite representing only 65.4% of total pathway usage, the MT‐Mucus pathway contributed to 81.8% of false positive errors, indicating reduced reliability when MM localization fails. Figure 5 represents the overall segmentation along with MM localization and sinusitis classification.

**TABLE 3:** Detection trigger method usage.

Image group	MM‐Mucus	MT‐Mucus
Left nasal cavity—positive	30	32
Left nasal cavity—non‐positive	1	2
Right nasal cavity—positive	6	31
Right nasal cavity—non‐positive	1	7
Overall	38 (34.6%)	72 (65.4%)

### 3.3 Clinical Deployment Feasibility

To evaluate the real‐time processing performance of our AI‐assisted sinusitis detection system, we measured the latency and throughput of image inference across the full test set. The system achieved an average latency of 0.073 ± 0.218 s per image, corresponding to an average frame rate of 22.2 frames per second (FPS). The observed latency jitter, defined as the standard deviation of latency across all processed images, was 0.218 s. These results confirm that our YOLOv11‐nano‐based model operates well within real‐time constraints for clinical video playback (≥ 20 FPS), enabling near‐instantaneous feedback in NE performance scenarios.

While frame rates exceeding 30 fps would be preferable, the achieved 22.2 fps performance meets clinical requirements for real‐time endoscopic guidance. The framework was implemented on NVIDIA Jetson Orin Nano edge computing hardware (1024‐core Ampere GPU, 6‐core Arm Cortex‐A78AE CPU, 8GB LPDDR5 memory, JetPack 5.1.2 with CUDA 11.4) providing 40 TOPS AI processing capability within a 7‐15 W power envelope. This edge computing approach ensures patient data privacy, reduces inference latency, and enables integration into existing clinical workflows without extensive infrastructure modifications, demonstrating practical feasibility for point‐of‐care diagnostic support systems.

## 4 Discussion

This study demonstrates that the ML framework can accurately localize relevant sinonasal landmarks and detect purulent mucus in NE images, which are key steps in the standardized assessment of sinusitis. The YOLOv11‑nano multi‑class segmentation model achieved F1 scores above 75% for all anatomical targets with the MT yielding particularly robust segmentation performance and the IT following closely. Although mucus detection and segmentation were intrinsically more challenging, the overall average F1 score of 86.9% underscores the framework's capacity to delineate anatomical landmarks and mucus in a manner consistent with annotation by a trained otolaryngologist.

When applied to an independent test set of CRSsNP cases, the aforementioned trigger pathway—relying primarily on the spatial overlap between segmented mucus and either the MM (MM‑Mucus) or MT (MT‑Mucus)—yielded an F1 score of 86.1% for left nasal cavity images. This level of diagnostic discrimination is on par with previously reported inter‑rater agreement by Bidwell et al. [14], suggesting that the ML framework effectively captures the visual cues that underpin expert decision‑making. Right nasal cavity performance was notably lower, likely reflecting known anatomical asymmetries, variable endoscope orientation, and occasional occlusion of the mid‑meatal region, which are issues that our fallback MT‑Mucus pathway partially addresses. For example, images of right and left nasal cavities were not matched for quality and symmetry. Anatomical variations such as septal deviations and spurs among the 50 patients chosen for the testing dataset would then unilaterally occlude relevant structures of the nasal cavity such as the MM and MT, thus preferentially limiting the model's ability to detect sinusitis on a particular side. The observed performance asymmetry between nasal sides reflects the complex interplay of anatomical variation, imaging factors, and algorithm design. The heavy reliance on MT‐Mucus fallback pathway for right‐sided diagnosis suggests that improving MM localization techniques could enhance bilateral performance consistency. The higher error contribution from the MT‐Mucus pathway suggests that direct MM visualization provides more reliable diagnostic information than the fallback approach of assessing mucus around the MT. This finding has important implications for algorithm deployment, as cases relying on MT‐Mucus triggering may warrant additional clinical scrutiny.

ROC analysis validates our threshold selection and demonstrates algorithm robustness across different operating points. The AUC of 0.762 indicates clinically relevant discriminative ability, comparing favorably with other medical imaging AI applications. The ability to adjust thresholds based on clinical context provides flexibility for diverse deployment scenarios, with lower thresholds favoring sensitivity for screening applications and higher thresholds optimizing specificity for confirmatory use. Although sensitivity and specificity can be further optimized—perhaps through incorporation of video‑based context or by refining classification/segmentation tasks including laterality—the present results validate the fundamental concept: that landmark‑based ML can implement core clinical best practices for sinusitis evaluation that align with current practice standards. The model's achieved overall 75.0% sensitivity and 76.0% specificity outperforms certain symptomatic and radiologic evaluation of CRSsNP demonstrated by Koskinen et al. (60% sensitivity for facial pain/pressure, and 38% SE for obstructed osteomeatal complex on CT) [15] and was on par with findings by Kim et al. in their meta‐analysis evaluating the usefulness of NE compared to sinus CT in patients with CRS (72.6% sensitivity and 76.7% specificity) [16]. A systematic review by Ebell et al. examining test accuracy for acute rhinosinusitis (0.83 sensitivity, 0.67 specificity on flexible endoscopy; 0.82 sensitivity, 0.38 specificity rhinoscopy with pus in the nasal cavity) [17] further underscores the potential clinical validity of the model's performance.

Equally important for real‑world adoption, our framework achieves an average inference latency of 0.073 ± 0.218 s per image, which translates to about 22.2 FPS on commodity hardware. This throughput clears the 20 FPS threshold required for real‐time video feedback and continues to validate the success of YOLO models determining landmarks in real as demonstrated by Ganeshan et al. [18] and Bidwell et al. [14], making it practically deployable for clinical use. Real‑time performance is especially relevant for primary care physicians (PCPs) and non‑specialists who may lack routine endoscopy experience because it capably provides instantaneous annotations of the MM and purulent mucus, which would bolster diagnostic confidence. Ultimately, this could pave the way to a reduction in unnecessary antibiotic prescribing in primary care settings as well as expedite referrals of appropriate cases to an otolaryngologist.

These results support the feasibility of integrating ML‑based landmark localization, mucus detection, and segmentation into standard endoscopic workflows. The system's segmentation accuracy combined with real‑time processing establishes a proof‑of‑concept for automated sinusitis assessment comparable to the diagnostic process of a trained expert. Future work will focus on expanding the dataset to include a broader spectrum of anatomic variants and disease severity and incorporating video‐based context for enhanced detection and accuracy.

This study's reliance on purulent mucus detection and segmentation limits its ability to detect non‐purulent mucus, which is crucial for diagnosing non‐bacterial sinus infections, allergic rhinitis, and other phenotypes of chronic sinusitis. Failing to identify non‐purulent secretions in the MM may lead to missed diagnoses of conditions like eosinophilic rhinosinusitis, fungal sinusitis, or subclinical inflammation, potentially delaying appropriate otolaryngologist intervention. MM localization presents inherent challenges due to anatomical variability, visualization constraints, and the limitations of geometric heuristics. The MM's narrow, often partially occluded nature requires inference from surrounding landmarks rather than direct visualization. Our rule‐based approach, while clinically informed, cannot fully capture the three‐dimensional spatial relationships and anatomical variations that expert clinicians intuitively assess. The differential pathway reliance observed between nasal sides directly reflects these MM localization challenges, with implications for diagnostic reliability in cases with suboptimal visualization or anatomical distortion. Laterality classification accuracy also directly impacts the overall model performance as errors in classification propagate through segmentation tasks, effectively multiplying their impact on final predictions. Instead of relying on computer vision to infer laterality, explicitly incorporating MT position as structured input can improve robustness and reduce ambiguity in left versus right nasal cavity identification. Additionally, in this paper, we used a heuristic method to select the MM area. This method is presumably less accurate than direct segmentation and may have to be replaced by other computer vision tasks that incorporate depth information and/or directly MM segmentation over the current heuristic.

While the overall results are promising, several important limitations must be acknowledged. The dataset was composed exclusively of images captured at a single center using a single imaging platform, predisposing the model to institutional and hardware‐specific biases. Moreover, the control group composition and demographic matching require further characterization to ensure balanced representation and minimize outside factors. External validation across multiple institutions, diverse patient populations, and varied endoscopic systems will be essential for establishing generalizability.

Performance asymmetries between left and right nasal cavities highlight the challenges of laterality classification and MM localization. These discrepancies likely reflect a combination of anatomical variability, endoscope orientation, and occlusion of key landmarks. Future research should focus on direct MM segmentation [19], potentially incorporating depth information rather than heuristic region inference. Similarly, explicitly modeling laterality rather than inferring it indirectly could reduce error propagation across downstream tasks.

Our framework currently relies on the RGB color space, which may limit cross‐platform compatibility across different monitors, lighting conditions, and endoscope systems. Exploring alternative color representations such as CIE‐LAB or HSV may improve tissue contrast and diagnostic consistency. Additionally, enhancing mucus detection to distinguish between purulent and non‐purulent subtypes will provide more granular diagnostic insights and better support treatment planning.

Subsequent work will include feasibility studies followed by prospective clinical trials to evaluate safety and efficacy in a clinical setting. Integrating polyp segmentation and mucus morphology detection, as demonstrated in prior work by Rampinelli et al. [20], Kwon et al. [21], and Gyawali et al. [22] will further expand diagnostic coverage. Finally, exploring hybrid architectures that combine YOLOv11's real‐time speed with the segmentation precision of U‐Net could advance both accuracy and usability, ultimately providing more precise and automated support for both otolaryngologists and non‐specialists.

## 5 Conclusion

This study presents the first validated ML framework for real‐time clinical‐decision support of sinusitis diagnosis using NE, achieving diagnostic performance comparable to trained otolaryngolgists while operating at clinically relevant speeds. By automating landmark identification and MM assessment—two critical yet subjectively variable components of endoscopic evaluation—this framework addresses the fundamental challenge of inter‐operator variability in sinonasal care. For practicing otolaryngologists, this technology offers a valuable diagnostic adjunct and teaching tool, while for non‐specialists, this could enable more confident initial assessments, potentially reducing inappropriate antibiotic prescriptions and ensuring timely specialist referrals.

## Conflicts of Interest

Edward D. McCoul is a consultant for 3D Matrix, Advanced Rx, Sanofi/Regeneron, Stryker and Zsquare.  The other authors have no conflicts of interest.
